# Digital anthropometry for body circumference measurements: Toward the development of universal three‐dimensional optical system analysis software

**DOI:** 10.1002/osp4.467

**Published:** 2020-11-06

**Authors:** Sima Sobhiyeh, Samantha Kennedy, Alexander Dunkel, Marcelline E. Dechenaud, Jerome A. Weston, John Shepherd, Peter Wolenski, Steven B. Heymsfield

**Affiliations:** ^1^ Metabolism‐Body‐Composition Pennington Biomedical Research Center LSU System Baton Rouge Louisiana USA; ^2^ Department of Mathematics Louisiana State University Baton Rouge Louisiana USA; ^3^ Cancer Center University of Hawaii Honolulu Hawaii USA

**Keywords:** anthropometry, nutritional assessment, three‐dimensional optical imaging, waist circumference

## Abstract

**Background/Objective:**

Digital anthropometric (DA) assessments are increasingly being administered with three‐dimensional (3D) optical devices in clinical settings that manage patients with obesity and related metabolic disorders. However, anatomic measurement sites are not standardized across manufacturers, precluding use of published reference values and pooling of data across research centers.

**Subjects/Methods:**

This study aimed to develop universal 3D analysis software by applying novel programming strategies capable of producing device‐independent DA estimates that agree with conventional anthropometric (CA) measurements made at well‐defined anatomic sites. A series of technical issues related to proprietary methods of 3D geometrical reconstruction and image analysis were addressed in developing major software components. To evaluate software accuracy, comparisons were made to CA circumference measurements made with a flexible tape at eleven standard anatomic sites in up to 35 adults scanned with three different commercial 3D optical devices.

**Results:**

Overall, group mean CA and DA values across the three systems were in good agreement, with ∼2 cm systematic differences; CA and DA estimates were highly correlated (all *p*‐values <0.01); root‐mean square errors were low (0.51–3.27 cm); and CA‐DA bias tended to be small, but significant depending on anatomic site and device.

**Conclusions:**

Availability of this software, with future refinements, has the potential to facilitate clinical applications and creation of large pooled uniform anthropometric databases.

**Abbreviations**
CA: conventional anthropometry
DA: digital anthropometry

## 1 INTRODUCTION

Body size and shape information provides valuable insights into a wide range of topics related to human obesity.^1^, ^2^, ^3^, ^4^, ^5^ Anthropometric measurements, such as circumferences that define body size and shape, are inexpensive and safely acquired for evaluating the health and nutritional status of patients with overweight and obesity across the full lifespan.^2^, ^3^ The use of these estimates, applied worldwide in highly varied settings, are advocated by numerous scientific organizations and health‐related associations as a means of weight trajectories, gauging the risk of developing chronic diseases, and many other topics of clinical and research interest.^5^ Moreover, anthropometric measurements defining body size and shape go beyond applications in patients with overweight and obesity and are widely applied in many other clinical nutrition areas.

An important application of conventional anthropometry (CA) is to quantify and monitor somatic features, such as adiposity level and adipose tissue distribution, as part of multicenter trials and survey protocols.^1^, ^6^, ^7^, ^8^ An ambitious goal would be to create large cloud‐based anthropometric databases by pooling the information collected in these studies and using them for numerous clinical and investigative purposes. Although building these global databases is a laudable objective, several roadblocks now limit the practicality of this approach. First, CA measurements are often highly variable between observers.^5^, ^9^ While implementing standardized protocols and using highly trained staff to perform assessments mitigates inter‐observer error, measurement bias from training and technique can still be pronounced. Additionally, even with the employment of competent technicians and staff, participant evaluations are time consuming and can effectively lead investigators to compromise the number and type of measurements to be collected as well as overall study sample size.^5^ Another concern is that conventional anthropometric measurements are typically transcribed by hand, thus increasing the risk of data entry errors.

Recent advances in three‐dimensional (3D) optical imaging technology are making large datasets possible by bypassing the inefficiency of developing similar resources using CA measurements. 3D scanners ranging from research‐grade laser systems to small in‐home devices^10^, ^11^, ^12^, ^13^ are increasingly popular and widely used. These scanners automatically give consumers and investigators hundreds of digital anthropometric (DA) measurements with little test–retest variation.^10^, ^11^, ^12^, ^13^, ^14^ However, through their proprietary software, each manufacturer provides DA measurements unique to their own system and the anatomic site definitions of these measurements are often unclear. Thus, pooling DA data acquired from different systems is largely precluded by variable anthropometric between‐scanner estimates for the same anatomic region. Consequently, a universal definition is required for obtaining DA measurements from different scanners despite their inherent design differences. An important consideration in establishing appropriate DA measurements is to find definitions that would best match standard CA measurements.

The process of comparing digitally and traditionally acquired body measurements is challenging due to the intrinsic nature in which each method identifies landmarks. For example, CA relies on palpation for anatomic structures to locate measurement sites, whereas DA is limited to superficial information to define measurement locations. As the relationship between standard CA measurements and scanner‐specific DA measurements is not available, DA methods might not be usable for health risk prediction from published models that, for example, rely on waist circumference estimates.^10^, ^15^

This study aimed to develop “universal” 3D analysis software with a two‐fold purpose: to provide clinicians a tool for gathering device‐independent standardized anthropometric body dimensions for estimating body composition and heath‐risks; and to initiate creation of large pooled anthropometric research‐oriented datasets. The developed universal software tool was evaluated by comparing DA measurements to corresponding CA measurements acquired at well‐defined and accepted anatomic locations acquired with a flexible tape by an expert anthropometrist.

## 2 MATERIALS AND METHODS

### 2.1 Participants

A convenience sample of healthy adults (age ≥18 years) at or above a body mass index of 18.5 kg/m^2^ were asked to arrive at the Pennington Biomedical Research Center Metabolism‐Body Composition Laboratory and change into form fitting shorts and in addition, if female, a sports bra. Subjects with hair that extended below their chin were asked to wear a swim cap. Prior to enrollment, all participants provided their written and informed consent. The experimental procedures were approved by the Pennington Biomedical Institutional Review Board as part of the larger Shape Up! Adults study which is publicly listed on ClinicalTrials.gov as ID NCT03637855.

### 2.2 Experimental design

The study was conducted in two phases. First, a team of engineers at Louisiana State University (Baton Rouge, LA) led by the authors stepped through the key stages of software development, solving technical problems at each stage. Following completion of the prototype software, participants underwent scanning by three commercially available 3D optical scanners as well as CA measurements collected by a highly trained staff member. The universal software was then used to analyze the images acquired by each scanner and generate estimates designed to match those of standard anthropometric definitions. These digital measurements produced by the universal software for each scanner were then compared to those acquired by CA.

### 2.3 Anthropometry

Accuracy and precision of measurement methods were evaluated as part of the parent Shape Up! Adults study.^11^, ^12^

### 2.4 Conventional

Conventional anthropometric measurements were made on each participant by a single highly trained staff member using a calibrated flexible tape. Circumferences of the chest, waist, hip, upper arms, thighs, calves, and ankles were measured and recorded in triplicate to the nearest 0.1 cm; results were averaged. Replicate measurements differing more than 0.5 cm were discarded and remeasured three additional times. For a description of measurement sites and associated references, see Table 1.^16^, ^17^ The coefficient of variation for the CA circumference measurements at our center range from 0.3%–0.9% for repeated measurements.^10^, ^11^, ^13^

**TABLE 1:** Conventional anthropometric circumference measurement sites^a^

Measurement Site	Definition
Chest	Participant stands erect with arms abducted to permit passage of the tape around the chest. The arms are lowered to the natural position once the tape is in place. In men the circumference is measured superficial to fourth intercostal space; in women, the circumference is measured at the maximum extension of the breast parallel to the floor
Waist	A mark is made at the lateral boarder of the right ilium identified through palpation. Waist circumference is measured at this point with the flexible tape remaining level with the floor
Hip	The circumference is measured at the maximum extension of the buttocks parallel to floor as the participant stands with feet together
Upper arm	The circumference is measured at the midpoint between the acromion process and the tip of the elbow as the participant stands palm up with arm positioned at a 90‐degree angle and upper arm pressed against the body
Thigh	As the participant stands with their knee supported at a 90‐degree angle, a mark is made at the midpoint between the inguinal crease and the proximal border of the patella. The circumference is measured at this point perpendicular to the femur
Calf	The tape is positioned horizontally around the calf and moved up and down to locate the maximum circumference in the plane perpendicular to the long axis of the calf
Ankle	Measured in the barefoot participant standing on a flat surface with feet slightly separated. Circumference taken at the level of the narrowest point of the ankle, perpendicular to the long axis, just proximal to the malleoli

^a^ All circumferences were collected with the participant standing between the technician and a mirror to ensure the tape remained parallel with the floor. Details of the measurement protocols and anatomic sites can be found in references #16 and #17. The coefficient of variation for these circumferences at our center ranges from 0.3%–0.9% for repeated measurements.^10^, ^11^, ^13^

### 2.5 Digital

Three commercial optical systems were used to obtain images of each participant. All three systems use similar inexpensive infrared technology for collecting depth information; however, each incorporates unique hardware features that result in slight variations in the user's experience. There are two variations in the technology, Time of Flight and structured light. Time of Flight technology calculates the distance between the camera and an object by measuring the time a projected infrared light takes to travel from the camera, reflect on an object, and then come back to the sensor. For the structured light technology, the camera emits a structure pattern on the surface of an object and observes the deformation of the pattern on the surface.

The Proscanner (Fit3D, Inc.) has three stationary cameras that are aligned vertically on a column. During image capture, the subject stands on a turntable and holds on to adjustable handles with arms held in a downward V position, the so‐called standard “A‐pose”. The cameras emit a structured light pattern that is distorted by the subject's figure and this deformation is used to calculate depth. Each scan takes approximately 40 s to complete.

The Styku (Styku LLC) scanner uses a single stationary camera on a tower connected to a rotating platform. The camera uses Time of Flight technology that calculates depth through phase shifts between emitted and reflected infrared light. The Styku scanner has a depth resolution of 512 × 424 pixels with a 70.6° × 60° field of view resulting in an average of 7 × 7 depth pixels per degree. To capture the image, the subject stands with his or her hands in a downward V as the turntable rotates for about 30 s. Unlike the Proscanner and SS20, the Styku scanner does not have handles for the subject to hold.

The SS20 (Size Stream LLC) uses 20 sensors that operate using the structured light method. The cameras are positioned at five different heights along four vertical columns surrounding the field of view. The subject remains stationary in the center of the columns holding on to handles with their arms in a downward V position. Image capture takes approximately four seconds to complete.

Previously published studies implementing these devices show precision estimates ranging from 0.3% to 5.0% for repeated measurements of the same 11 circumferences.^10^, ^11^, ^13^

### 2.6 Universal software development

The software program Matlab (Mathworks) was used to create a prototype of the universal software that consists of a three‐step procedure: preprocessing, landmark detection, and DA calculation. The investigators will make the Matlab version of the software freely available without restrictions upon request. A more general‐use version of the software is in development.

#### 2.6.1 Preprocessing

**FIGURE 1:**
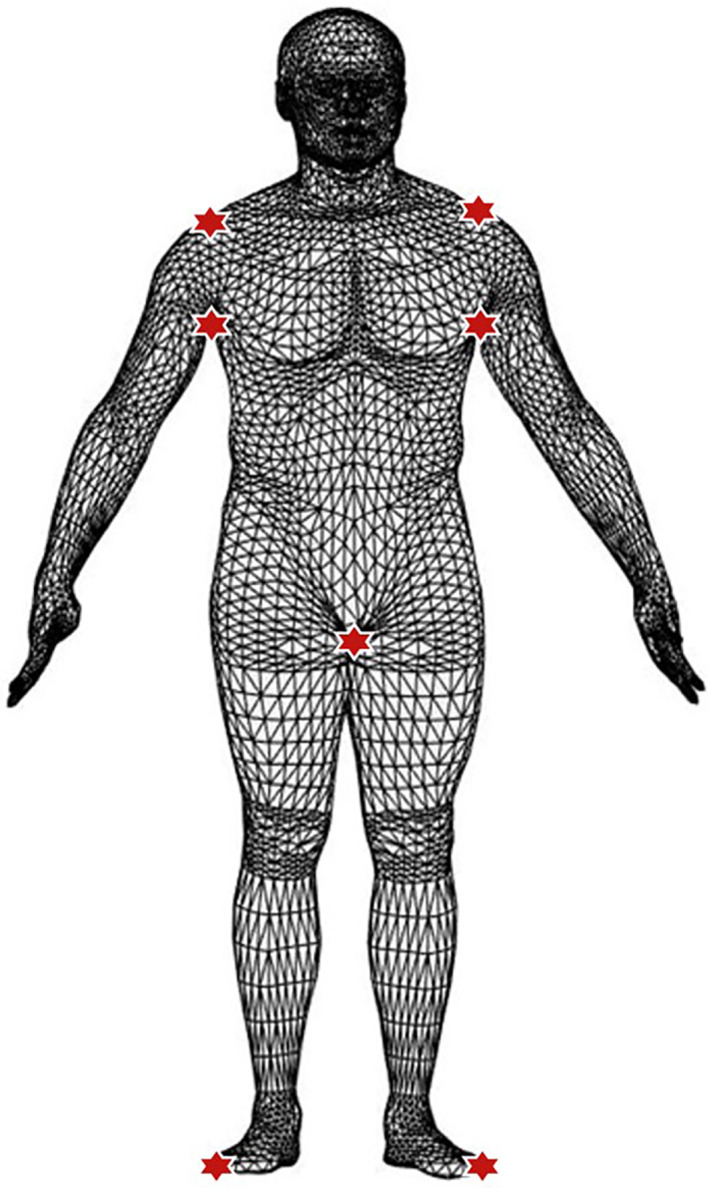
Avatar image of the input triangular mesh used by the universal software. The 3D scans are initially in a triangular mesh format generated directly by the proprietary software of each scanner. The scanners generate a unique surface composed of small planar triangular faces, and these faces all connect to create the 3D surface. The “definition” of each scan is thus a list of “faces” and a list of “vertices”. The seven landmarks detected by the universal software are shown as red stars. Detecting the Crotch In Mesh Analysis is an algorithm developed in an earlier study^17^ for accurately detecting the crotch and armpit landmarks. The right and left shoulders are identified as the highest vertex directly above the right and left armpits whereas the feet are identified as the lowest vertices on the right and left sides of the body

The data for each 3D scan contains a triangular mesh similar to the one shown in Figure 1, where the 3D cloud points are represented by a list of vertices and meshing is represented by a list of “faces”. To ensure comparable analysis results across devices, the developed software initially reformats and repairs the cloud points and the mesh such that the same software can be used for the 3D data obtained from different scanners. This step consists of correcting mesh alignment, adjusting poorly shaped faces and faces causing meshing defects, and reconstructing the mesh where there is missing data known as “holes”. Scan Reconstruction for Anthropometry Measurements (ScReAM) software was used for reconstructing the mesh as reported in an earlier study.^17^, ^18^

#### 2.6.2 Landmark detection

After preprocessing, basic landmarks, represented by red stars in Figure 1, are detected on the avatar. These landmarks include the crotch and the left/right foot, armpit, and shoulder. The shoulder landmarks are the highest vertex directly above each armpit and the feet are identified as the lowest vertices on each side of the body. A previously published algorithm referred to as Detecting the Crotch In Mesh Analysis (DeCIMA)^17^ was used for identifying accurate locations of the crotch and armpits. A detailed description of DeCIMA can be found in an earlier report.^17^

### 2.7 Calculating DA measurements

**FIGURE 2:**
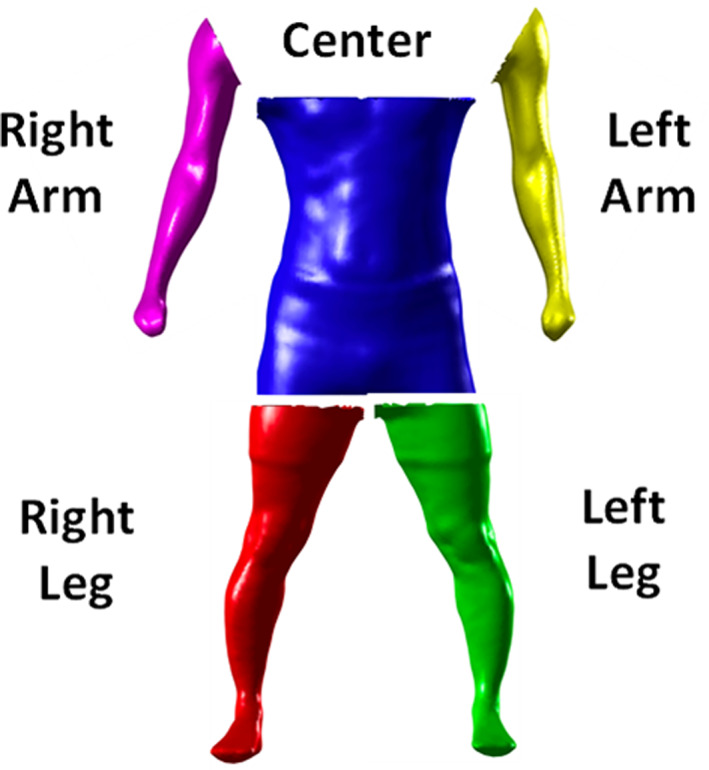
Partitioning of optical scans into left/right arm, left/right leg, and center body. Arms are segmented from the center body by a line connecting the shoulder and armpit landmarks shown in Figure 1. Legs are segmented from center body at a horizontal plane that crosses the crotch landmark, also shown in Figure 1. The center body is the area partitioned by horizontal lines crossing the armpit and crotch landmarks

Using the landmarks as points of reference, each scan is first segmented into arm, leg, and center segments (Figure 2). Then each body partition is cross sectioned at the locations where circumferences are defined. For the center segment, the cross‐sections are along the transverse axis. For the arm and leg segments, the cross‐sections are along the arm and leg; that is, the cross‐sections are not parallel to the floor but rather parallel to the arm and leg. That is, if an axis is created along the arms and legs, that axis would be at an angle (roughly 45°) with a reference to the longitudinal axis of the body. The cross section of the arms and legs are then taken perpendicular to the axis defined along the arms and legs, and not perpendicular to the longitudinal axis. Visual diagrams of where circumferences, including the chest, hip, waist, upper arms, thighs, calves, and ankles are digitally estimated can be obtained by request to the authors. Similar images are provided in references #16 and #17. A detailed explanation of the algorithm implemented to automatically create the cross‐sections as well as define and estimate circumferences can be found in previously published work.^17^

### 2.8 Statistical methods

Digital measurements calculated by the universal software using images from each of the three commercial 3DO systems were compared to corresponding flexible tape reference measurements. Statistical analyses, graphs, and tables were generated using JMP Pro 15.0.0 2019 (SAS Institute Inc.) and Microsoft Excel 2016 (Microsoft Corp.). Of the total participant sample, 18 were evaluated on the SS20 scanner due to instrument availability. Scans were completed on all 35 participants with the Proscanner and Styku systems. The anonymized data set necessary to replicate the current study findings can be obtained by request from the authors.

To explore the level of agreement between the universal software‐derived DA measurements and their CA counterparts, the following were tested for each scanner: (1) the magnitude and significance of between‐method CA‐DA differences (Δ*s*) using paired *t*‐tests for each of the eleven circumferences, (2) the magnitude of correlations between DA and CA measurements using simple linear regression analysis, and (3) if between‐method DA‐CA bias was present as quantified using Bland–Altman analyses. Paired, two‐sided *t*‐tests were used to compare DA circumference estimates derived from the universal software using images obtained from each device to corresponding CA measurements made with the flexible tape. Mean differences at *p* < 0.05 were considered statistically significant. For linear regression and Bland–Altman analyses, significance was set at *p* < 0.05.

## 3 RESULTS

The multiethnic sample included 35 adult participants, 12 females and 23 males, with mean (±SD, range) weight (64.1 ± 17.2 kg; 40.8–120.2 kg), height (172 ± 4 cm; 160–182 cm), and body mass index (21.6 ± 5.2 kg/m^2^; 15.0–39.1 kg/m^2^).

### 3.1 Algorithm evaluation

Circumference measurement means (±SD) for CA and DA along with their mean differences (Δ*s*) are presented in Table 2. Overall, the group mean values for circumferences evaluated by CA and DA were similar with small but statistically significant differences that varied by system and anatomic site. Absolute mean CA‐DA differences were about 2 cm, except for a few outliers, across all three systems and eleven anatomic sites. The mean systematic differences between CA and DA were negative for Styku and positive for Proscanner and SS20. As the mean absolute differences (∼2 cm) were similar across anatomic sites, percentage CA‐DA differences were relatively small for the large chest, waist, and hip measurements (i.e., ∼2 cm for sites varying in circumference from ∼85–100 cm or ∼2%–3%) and larger for the small arm (i.e., ∼2 cm for sites varying circumference from ∼30–35 cm or 5%–7%) and ankle measurements (i.e., ∼2 cm for sites with circumferences of ∼20–25 cm or 8%–10%).

**TABLE 2:** Results of CA and DA circumference measurements

	CA	Styku	Δ Mean (Styku)	ProSc	Δ Mean (ProSc)	SS20	Δ Mean (SS20)
	*n* = 35	*n* = 35		*n* = 35		*n* = 18	
Chest	91.7 ± 10.9	91.1 ± 9.6	−0.6	94.7 ± 8.7	3.0*	97.7 ± 10.4	5.2*
Waist	84.2 ± 13.9	78.5 ± 14.5	−5.7*	86.8 ± 14.8	2.5*	87.9 ± 15.3	2.9^Ŧ^
Hip	98.9 ± 8.8	96.0 ± 8.9	−2.9*	100.3 ± 8.8	1.3*	103.0 ± 9.9	1.9*
Upper arm							
Right	29.34 ± 5.36	25.8 ± 4.7	−3.5*	30.4 ± 4.6	1.0^Ŧ^	33.4 ± 5.5	3.1*
Left	29.0 ± 5.52	26.1 ± 4.8	−2.9*	31.4 ± 4.9	2.4*	33.6 ± 6.1	3.2*
Thigh							
Right	52.8 ± 5.8	50.7 ± 5.1	−2.1^Ŧ^	53.9 ± 4.8	1.1^Ɨ^	55.2 ± 5.9	1.0
Left	51.9 ± 5.9	50.7 ± 5.3	−1.2^Ŧ^	53.9 ± 4.5	2.0*	55.6 ± 6.1	2.2^Ŧ^
Calf							
Right	36.3 ± 3.5	33.1 ± 3.2	−3.2*	37.7 ± 3.5	1.5*	37.9 ± 4.3	1.0*
Left	36.0 ± 3.6	33.4 ± 2.9	−2.6*	38.5 ± 3.3	2.4*	38.0 ± 4.3	1.3*
Ankle							
Right	21.8 ± 2.0	15.9 ± 1.9	−5.9*	24.8 ± 2.2	3.0*	23.6 ± 2.6	1.5*
Left	21.8 ± 2.1	16.0 ± 2.2	−5.8*	24.0 ± 2.3	2.2*	24.2 ± 2.6	2.1*

*Note*: Results are in cm, mean ± SD. Δ is calculated as DA‐CA. Styku and Proscanner, *n* = 35; SS20, *n* = 18.

Abbreviations: CA, conventional anthropometry; DA, digital anthropometry; ProSc, Proscanner.

*p < 0.0001, ^Ŧ^ *p* < 0.01, ^Ɨ^ *p* < 0.05.

The system root‐means square errors and linear regression analysis results are presented in Table 3 with corresponding Bland‐Altman analyses. The root mean square error ranged from about 1 to 3 cm with a tendency for greater prediction errors in regression models evaluating results from Styku system images against CA. The linear correlations between CA and DA overall had high *R* ^2^ values, most of which were greater than 0.90 (*p* < 0.001), although there were a few exceptions. Notably, the *R* ^2^s observed for thigh measurements estimated from Styku images were lower (right, 0.77; left, 0.89) and even lower for calf (0.62 and 0.77) and ankle (0.21–0.37) measurements, although all regression analyses remained statistically significant (*p* < 0.05 to <0.001). The sources of these between‐instrument difference are reviewed in the Discussion.

**TABLE 3:** Correlations between conventional and digital circumference measurements

		RMSE	*R* ^2^	Bland–Altman Analysis	
				*R* ^2^	Slope
Chest	Styku	2.31	0.94*	0.24^Ŧ^	0.13
	ProSc	1.64	0.97*	0.60*	0.22
	SS20	2.49	0.95*	0.41^Ŧ^	0.19
Waist	Styku	3.27	0.95*	0.04	−0.04
	ProSc	2.54	0.97*	0.10^Ɨ^	−0.06
	SS20	2.60	0.97*	0.05	−0.04
Hip	Styku	1.93	0.95*	0.01	−0.02
	ProSc	1.31	0.97*	0.01	−0.06
	SS20	1.34	0.97*	0.03	−0.05
Upper arm					
Right	Styku	1.37	0.92*	0.18^Ŧ^	0.14
	ProSc	1.05	0.95*	0.31^Ŧ^	0.15
	SS20	1.19	0.96*	0.15	0.09
Left	Styku	1.30	0.93*	0.20^Ŧ^	0.13
	ProSc	1.44	0.92*	0.13^Ɨ^	0.11
	SS20	0.99	0.97*	0.01	0.01
Thigh					
Right	Styku	2.49	0.77*	0.07	0.14
	ProSc	2.03	0.83*	0.19^Ŧ^	0.21
	SS20	1.82	0.91*	0.15	0.13
Left	Styku	1.78	0.89*	0.09	0.12
	ProSc	1.41	0.91*	0.43*	0.27
	SS20	1.79	0.92*	0.16	0.13
Calf					
Right	Styku	2.04	0.62*	0.02	0.10
	ProSc	0.57	0.97*	0.07	0.05
	SS20	0.51	0.99*	0.04	0.02
Left	Styku	1.41	0.77*	0.16^Ɨ^	0.22
	ProSc	1.00	0.91*	0.07	0.08
	SS20	0.57	0.98*	0.15	0.05
Ankle					
Right	Styku	1.75	0.21^Ŧ^	0.00	0.06
	Fit3D	1.01	0.79*	0.03	−0.08
	SS20	0.67	0.94*	0.00	0.00
Left	Styku	1.75	0.37*	0.01	−0.07
	Fit3D	0.74	0.90*	0.10	−0.11
	SS20	0.64	0.94*	0.02	−0.04

*Note*: Styku and ProSc, *n* = 35; SS20, *n* = 18.

Abbreviations: CA, conventional anthropometry; DA, digital anthropometry; ProSc, Proscanner; RMSE, root mean square error.

^Ɨ^ *p* < 0.05, ^Ŧ^ *p* < 0.01, *p < 0.001.

Bland‐Altman analyses revealed significant bias in 11 of the 33 evaluations with the highest observed slopes comparing CA and DA results derived from the Proscanner system.

**FIGURE 3:**
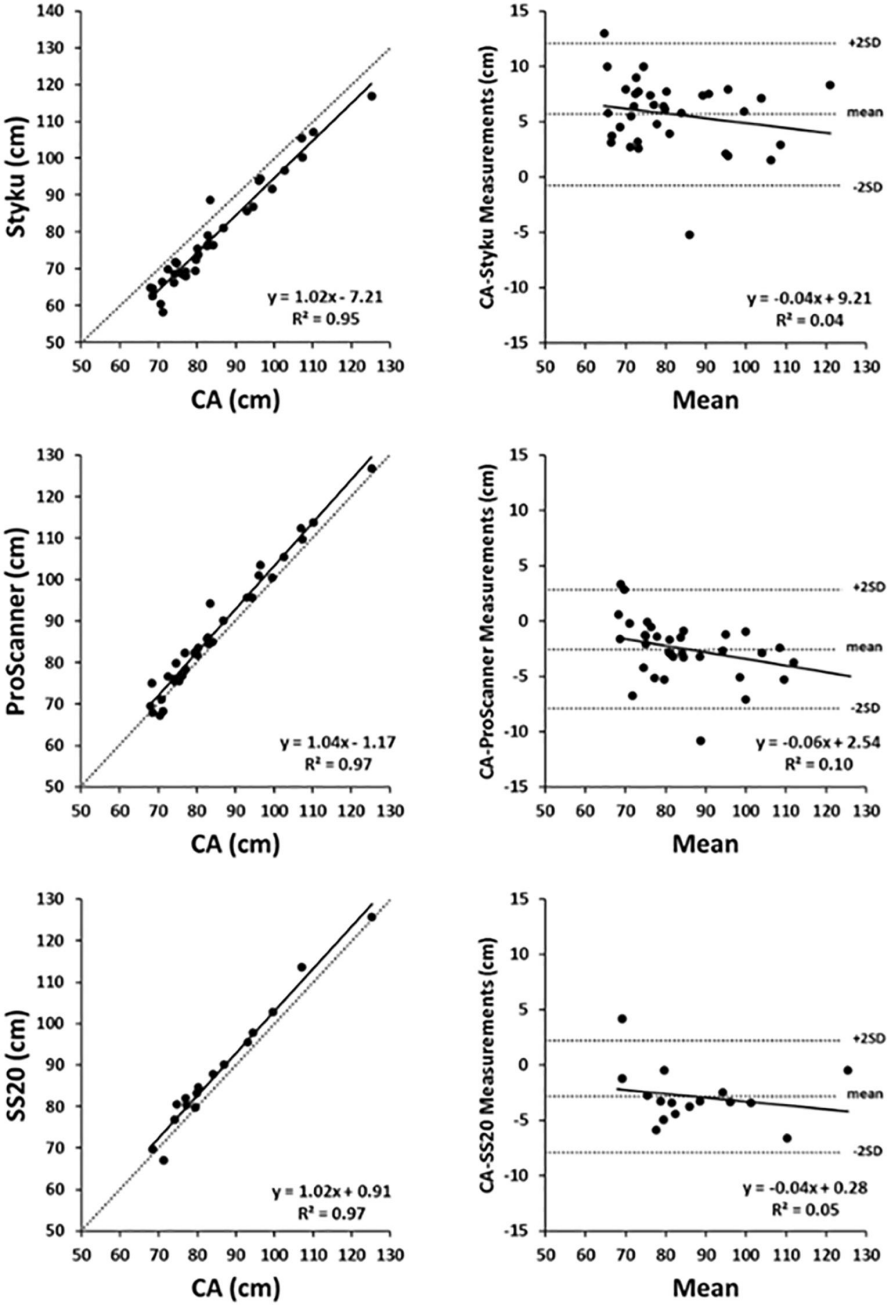
Scatter plots of digital versus conventional anthropometric estimates of waist circumference and associated Bland‐Altman plots. The regression equations and corresponding *R* ^2^s are shown in each panel (all *p* < 0.001) along with the line of identity (dashed line). The Bland–Altman plots horizontal lines are the mean CA‐DA difference ± 2 SDs. Additional details of these analyses are given in Table 3. CA, conventional anthropometric; DA, digital anthropometric

Representative waist circumferences estimated by the universal software from images collected on each 3D optical system (Styku, Proscanner, and SS20) plotted against CA measurements are shown in Figure 3 along with the corresponding Bland‐Altman plots. Correlations between CA and DA waist circumference estimates had *R* ^2^s ranging from 0.95 to 0.97 (*p* < 0.001); measurement bias was only significant for DA estimates from the Proscanner (*p* < 0.05).

## 4 DISCUSSION

The current study documents the development pathways and initial validation of software that processes images acquired on commercially available 3D optical devices and produces a standardized output of anthropometric body dimensions. These standardized anthropometric measurements can be used for a wide range of clinical and research purposes. The current approach overcomes the limitations of pooling arbitrarily defined body measurements rendered by proprietary systems, thus providing clinicians and investigators with a means of aligning a patient's DA results with those reported by national and international agencies and to merge data across different 3D devices. Construction of cloud based mega databases that house these kinds of standardized measurements creates an opportunity to develop prediction equations and models on an global scale with samples potentially ranging in thousands or even millions.^19^ Our group is now developing a cloud‐based system that will actualize this concept in collaboration with an international team of investigators.

A key feature of the universal software is that it uses identical anatomic landmarks in defining body circumferences independent of device or manufacturer. The selected landmarks match those used by national and international organizations in conducting health surveys such as NHANES. Even with programming efforts, small and sometimes systematic CA‐DA measurement differences were observed. These differences arise because of scanner‐specific features that define the original avatar's shape that's generated by the instrument's proprietary software. One predictable cause of these effects is that the alignment between the CA estimates and automatically identified DA measurements was imperfect. The errors from these small circumference differences can propagate when taking ratios such as those of the waist and hip. With additional analyses, these kinds of measurement differences can be compensated for in future software iterations. As these CA‐DA differences were variable across systems, other strategies may be needed such as performing human or phantom “calibration” studies for each new system introduced to the market. This approach is well recognized with other devices that are used as part of multicenter research or clinical programs. One vision is to include scanner‐specific adjustments to data stored in cloud sites, thereby automatically making the minor compensations needed to achieve perfect alignment between commercial devices.

Another cause for CA‐DA differences involves specific features of the evaluated participants or with device hardware. Of human anatomic features, errors can arise from the difficulty in finding the crotch and armpit landmarks that help the software navigate through different body parts. In the case of participants with obesity, detecting these landmarks is challenging as legs may not be clearly separated in the thigh region and the arms and trunk also can touch each other making it hard to separate the two automatically.

Advanced algorithms were introduced to address these challenging cases.^17^, ^18^ These approaches resulted in improved crotch and armpit detection accuracy, although armpit detection in people who are overweight and obese remains an open challenge. One solution would be to capture scans with subjects standing in the T‐pose rather than A‐pose position or to use post‐imaging reposing software to reconfigure A‐pose to T‐pose scans. Substantial improvements have been observed in the arm measurements obtained by the universal software after converting scans into the T‐pose in a follow up unpublished pilot study.

The armpit and crotch are also the regions that often have “holes” in the captured 3D surface. The algorithm for patching these and other regional holes was developed so that the mesh patches follow the curvature of the body and thus do not compromise body shape. Moreover, these areas are not the sites at which DA measurements are made and thus their influence on accuracy is minimal.

The number of cameras and their position appeared to impact the devices' accuracy in measuring the lower extremities. The Styku system incorporating one stationary camera showed considerably more visible distortions around the calves and ankles compared to the systems with multiple, vertically aligned depth cameras such as the Proscanner and SS20. Although ankle circumferences matched between SS20 and Proscanner and data from both scanners matched with the corresponding CA measurements, the ankle circumferences measured from the Styku scans did not match with either of these devices or with the CA measurements. The slopes of Bland–Altman plots also showed similar outcomes: Bland–Altman slopes comparing ankle circumferences measured on the SS20 and Proscanner were close to zero, while comparing ankle circumferences measured on either of these scans with Styku scans, the Band–Altman slopes were larger.

The effect of Styku's single stationary camera on the observed mean DA‐CA differences can also be observed when moving distally along the leg. The mean difference between DA and corresponding CA measurements increases from 1–2 cm at the thighs to 2–3 cm at the calves, and then to 6 cm at the ankles. By contrast, for both Proscanner and SS20, the mean difference between DA and corresponding CA measurements at the thighs, calves, and ankles are all in the same range (1–3 cm) with no increasing pattern moving down along the legs.

The Proscanner and Styku systems employ rotating platforms that reduce the need for multiple cameras surrounding the subject. To complete a scan, the participant poses on the platform and remains still while the device completes a full revolution in under one minute. By contrast, the SS20 has 20 cameras and does not rotate the participant and captures the 3D image in only 4 s. The left and right sides of the SS20 scans are acquired simultaneously under similar conditions, reducing noise and avatar deformation caused by intra‐scan movement. This fundamental scanner difference may account for the slightly lower R^2^s of left and right‐side measurements from the Proscanner in comparison to the SS20 (Table 2).

Intra‐scan movement was consistently less pronounced when device features helped stabilize the participant. For example, more movement and image distortion were apparent in scans from the Styku system that has a turntable but no handlebars. These movement artifacts have been observed in other studies^10^, ^11^; however, these artifacts rarely occurred in the present study with the systems that provided handles (Proscanner) or that had minimal scan times and remained stationary (SS20). While handles produced more consistent results at distal extremities, they also obstructed light waves in such a way that over exaggerated the size of the user's wrists and forearms.

Although the results reported in this study are adequate to support the initial use of the proposed universal software and its algorithms, a larger sample of subjects is required to better analyze and compare different scanning technologies. Considerably larger and more diverse samples will also allow for further examination of variables such as total scan time and how the time difference between left and right‐side image capture with rotating platform systems influences scan quality. The current study identified these device hardware limitations and next generation systems can be designed to overcome these sources of measurement error.

Another concern is that flexible tape measurements were used as the reference to evaluate the accuracy of DA measurements. However, CA measurements contain human error and can be difficult to measure accurately for more robust or curvy body shapes. While precision estimates are inherently low due to measuring protocols in place preventing larger deviations, this does not account for measurement accuracy. High resolution laser‐based scanners might serve as an alternative to potentially less accurate CA in future studies. However, these devices tend to be costly and may not be practical for use in large scale trials carried out at multiple research centers.

This study validated the proposed universal software for three optical imaging devices (Styku, Proscanner, and SS20). In the next step, the plan is to extend the application of the developed software to scans obtained by other devices, specifically those optimized for home use. Due to compromises in hardware size and quality to make these devices affordable, scans usually contain more image artifacts. To ensure the universal software works for all devices, the future plan is to train a Convolutional Neural Network (CNN) with the results obtained by the three evaluated devices. Once trained, the CNN can be tested for producing similar results on other devices.

In conclusion, universal 3D optical scan analysis software was developed and critically evaluated in the current study. The software takes a 3D optical scan in the form of a triangular mesh, first reformats and edits the mesh, then automatically detects landmarks such as the feet, armpits, shoulders and crotch, and lastly calculates DA measurements including chest, hip, waist, mid‐upper arm, thigh, calf, and ankle circumferences. The software provides standard definitions for DA measurements that are not only manufacturer‐independent, but also closely match CA measurements in practice. Differences between CA and DA were detected, and areas of software and device hardware improvement have been identified. With further software updates and refinements, clinicians who acquire data with 3D optical devices and then process the data with universal software will then be able to reference patient results against published normative values. These developments moving forward will open a path to creating large anthropometric datasets as standardized data can be collected across centers using different 3D optical devices. Such large datasets with diverse samples will create an opportunity for conducting new studies in a wide range of health and fitness topics.

## CONFLICT OF INTEREST

The authors and their close relatives and their professional associates have no financial interests in the study outcome, nor do they serve as an officer, director, member, owner, trustee, or employee of an organization with a financial interest in the outcome or as an expert witness, advisor, consultant, or public advocate on behalf of an organization with a financial interest in the study outcome.

## AUTHOR CONTRIBUTIONS

Sima Sobhiyeh, Peter Wolenski, Alexander Dunkel, Marcelline E. Dechenaud, and Jerome A. Weston designed research; Sima Sobhiyeh, Samantha Kennedy, Jerome A. Weston, Marcelline E. Dechenaud, John Shepherd, and Steven B. Heymsfield conducted research; Sima Sobhiyeh, John Shepherd, and Steven B. Heymsfield provided essential materials; Sima Sobhiyeh, Samantha Kennedy, Marcelline E. Dechenaud, Peter Wolenski, and Steven B. Heymsfield analyzed data; Sima Sobhiyeh, Samantha Kennedy, Marcelline E. Dechenaud, John Shepherd, and Steven B. Heymsfield wrote paper; Sima Sobhiyeh, Samantha Kennedy, Alexander Dunkel, Marcelline E. Dechenaud, Jerome A. Weston, John Shepherd, Peter Wolenski, and Steven B. Heymsfield had primary responsibility for final content.
